# Uranium Exposure, Hypertension, and Blood Pressure in the Strong Heart Family Study

**DOI:** 10.5888/pcd22.240122

**Published:** 2025-04-24

**Authors:** Kevin P. Patterson, Abigail Onderwyzer Gold, Miranda J. Spratlen, Jason G. Umans, Amanda M. Fretts, Walter Goessler, Ying Zhang, Ana Navas-Acien, Anne E. Nigra

**Affiliations:** 1 Department of Environmental Health Sciences, Columbia University Mailman School of Public Health, New York, New York; 2 Vagelos College of Physicians and Surgeons, Columbia University, New York, New York; 3 MedStar Health Research Institute, Washington, District of Columbia; 4 Georgetown–Howard Universities Center for Clinical and Translational Science, Washington, District of Columbia; 5 Department of Epidemiology, University of Washington, Seattle; 6 Institute of Chemistry, Karl-Franzens University of Graz, Graz, Austria; 7 The University of Oklahoma Health Sciences Center, Oklahoma City

## Abstract

**Introduction:**

Uranium is common in drinking water, soil, and dust in American Indian communities. Hypertension is a cardiovascular risk factor affecting American Indians. We evaluated the association between uranium exposure and incident hypertension and changes in blood pressure among Strong Heart Family Study participants.

**Methods:**

We included 1,453 participants ≥14 years with baseline visits in 1998–1999 or 2001–2003, and follow-up in 2001–2003 and/or 2006–2009. We estimated the association of urinary uranium with changes in systolic and diastolic blood pressure levels over time and hypertension incidence; we accounted for family clustering.

**Results:**

Median (IQR) baseline urinary uranium levels were 0.029 (0.013–0.059) μg/g creatinine; 17.4% (n = 253) of participants developed hypertension. In the comparison of the urinary uranium quartile 4 (highest concentration) and quartile 1 (lowest concentration), the multi-adjusted risk ratio (95% CI) of incident hypertension was 1.44 (1.04–1.99). The associations between urinary uranium with changes in systolic and diastolic blood pressure were null and nonlinear, respectively. Both associations were modified by study site, and diastolic blood pressure showed a positive association beyond 5 µg/g creatinine. The association between urinary uranium and change in systolic blood pressure was inverse in Arizona and Oklahoma, and positive in North Dakota/South Dakota at higher ends of the uranium distribution.

**Conclusion:**

Findings suggest a higher risk for hypertension at uranium levels typical of the Southwest and Great Plains than at levels in other regions (<0.01 µg/g creatinine); the associations with changes in systolic and diastolic blood pressure levels were consistent with a positive association with higher uranium exposure. Prospective research is critical to characterize the cardiovascular effects of uranium and develop preventive strategies for US Indigenous communities disproportionately exposed.

**Summary**

**What is already known on this topic?**

American Indian communities disproportionately experience elevated exposure to uranium and a high prevalence of cardiovascular risk factors. Prior cross-sectional evidence suggests the two may be related but lacks sufficient representation from this population.

**What is added by this report?**

We leveraged the Strong Heart Family Study, the largest ongoing epidemiologic cohort of American Indians from the Great Plains and Southwest, to prospectively evaluate the associations between urinary uranium with hypertension and blood pressure measures. We found positive associations with increasing quartiles of urinary uranium levels.

**What are the implications for public health practice?**

Policy, primary, and secondary interventions should address inequities in uranium exposure via drinking water, diet, and dust, focusing on community education about relevant local environmental sources.

## Introduction

Uranium is a naturally occurring toxic metal commonly found in the western United States. Populations from several American Indian communities in the Southwest and Great Plains have shown, on average, higher metal levels in their urine compared with urban populations across the US (1–3). This disproportion might be explained by elevated levels of uranium in rocks and soil that lead to groundwater and surface water contamination in rural and suburban areas. Drinking water is a substantial source of uranium exposure in the US and is particularly relevant among rural and Native American populations, who rely more on private, unregulated water wells than on public sources in some areas (4). Both unregulated wells and public drinking water are major sources of total uranium exposure in American Indian communities (5). In many areas of the Southwest and Midwest, water wells exceed the US Environmental Protection Agency drinking water standard of 30 parts per billion (ppb) uranium in public drinking water supplies (6,7). Uranium groundwater contamination occurs naturally but is also exacerbated by a long history of uranium mining in these areas, with little to no clean-up (8). Most uranium mines are located on either federal or tribal land (9). For example, approximately 500 abandoned uranium mines are in the Navajo Nation, and an estimated 286,346 American Indians live less than 10 km from a mine (10). Climate change may also increase the mobilization of metals into groundwater (11,12), along with increased use of nitrate-containing fertilizer, which releases uranium stores (13).

The leading cause of death among American Indian people is cardiovascular disease (CVD), for which hypertension is a major risk factor (14). American Indian adults are 10% more likely than White adults to have high blood pressure (15). According to the Strong Heart Study (SHS), the prevalence of hypertension — defined as systolic blood pressure ≥140 mm Hg or diastolic blood pressure ≥90 mm Hg or use of antihypertensive medication — among American Indians aged 45 to 74 years was close to 50% at the Oklahoma and Arizona study centers and approximately 25% at the North Dakota and South Dakota study center at baseline (1989–1991) (16).

Uranium is known to cause kidney damage and cancer, but it is unclear if it also has implications for CVD — in particular, hypertension. In a mixture analysis in the 2011–2016 National Health and Nutrition Examination Survey (NHANES), urinary uranium was significantly associated with prevalent hypertension, which supports evidence indicating that uranium exposure can be a risk factor for hypertension (17). Previous studies of uranium workers showed that uranium exposure may be associated with angina, increases in deaths due to circulatory system disease, and hypertension or risk factors for the development of hypertension (18–20). On the Navajo Nation, uranium mining exposure was associated with hypertension, and further molecular evidence showed that physical proximity to abandoned uranium mines predicted endothelial transcriptional response to serums that included biomarkers of inflammation chemokine (C–C motif) ligand, vascular cell adhesion molecule-1, and intercellular adhesion molecule-1 (21,22). However, little is known about the relationship between uranium exposure among people with chronic low-level uranium exposure and incidence of hypertension and change in blood pressure over time. More research is needed to understand the effects of chronic low-level uranium exposure on CVD (23).

In this study, we examined the association of differential uranium exposure across the 3 centers of the Strong Heart Family Study (SHFS) with the incidence of hypertension and with blood pressure change during the follow-up period. The SHFS is a family expansion of the original SHS cohort, and it provides data on urinary uranium. We used urinary uranium levels as a marker of internal uranium dose. We hypothesized that after controlling for relevant sociodemographic and blood pressure risk factors, higher uranium exposure versus lower exposure, as determined in urine, would be associated with increased systolic and diastolic blood pressure levels and an increased risk of hypertension.

## Methods

The SHS is a population-based study of CVD in 12 participating American Indian communities in Arizona, Oklahoma, North Dakota, and South Dakota. Recruitment of men and women aged 45 to 74 years took place from 1989 to 1991. In 1998, the SHFS began; it was designed to study genetic and environmental determinants of diabetes and CVD among family members of the SHS (24). The investigators recruited 2,919 participants during 1998–1999 (Visit 3 pilot) and 2001–2003 (Visit 4), after excluding a community that declined participation in additional research. Participants recruited in 1998–1999 (n = 428) had follow-up visits in 2001–2003 and 2006–2009 (Visit 5). Participants recruited in 2001–2003 (n = 2,491) had a single follow-up visit in 2006–2009.

Hypertension is common among people with diabetes and is associated with renal dysfunction via mechanisms that include increased renal sodium reabsorption and endothelial cell dysfunction (25). We included young adult and adult participants who were free of diabetes at baseline and had sufficient urine available for uranium analyses (n = 1,948). We excluded 2 participants whose creatine-adjusted urinary uranium levels were greater than 10 times the 99th percentile. We also excluded participants missing information on relevant confounders at baseline, including education (n = 9), smoking status (n = 2), body mass index (BMI) (n = 7), systolic blood pressure (n = 1), diastolic blood pressure (n = 1), estimated glomerular filtration rate (eGFR) (n = 1), urinary cadmium (n = 25), or creatinine. We further excluded participants missing data on systolic and diastolic blood pressure at follow-up Visit 5 (n = 25) and prevalent hypertension cases at baseline (n = 422), making 1,453 participants available for this study. The SHS and SHFS protocols were approved by participating tribal communities and all institutional review boards (IRBs), including the IRBs of the Indian Health Service. All participants provided written informed consent, and all participating communities reviewed and approved this article.

### Urinary uranium measurements

Spot urine samples were frozen within 1 or 2 hours of collection and stored at −80 °C at Medstar Health Research Institute in Maryland. Detailed methods are described elsewhere (26). Urinary uranium concentrations were measured in spot urine collected at the baseline SHFS visit by using inductively coupled plasma-mass spectrometry with a multi-element protocol at the Trace Element Laboratory of Graz University, Austria (26).

To account for urine dilution, we divided urinary uranium concentrations by urinary creatinine concentrations (µg/g creatinine). The limit of detection (LOD) for uranium was 0.01 µg/L of urine (81.4% >LOD). All samples below the LOD were replaced by the LOD divided by the square root of 2. Urinary uranium was right-skewed and log-transformed for all analyses with a continuous predictor.

### Hypertension measurements

Blood pressure was determined by measuring brachial artery blood pressure (first and fifth Korotkoff sounds) 3 consecutive times with a mercury sphygmomanometer (WA Baum Co, Inc). Participants were seated and rested for 5 minutes before blood pressure measurements. The cuff was placed on the right arm, pulse occlusion pressure was determined, and the cuff was inflated to 20 mm Hg above that pressure. To estimate blood pressure, the mean of the last 2 measurements was used. Hypertension was defined as the use of antihypertensive medication, or a systolic blood pressure of ≥140 mm Hg or a diastolic blood pressure of ≥90 mm Hg. At baseline, by design for this study, none of the participants were taking antihypertensive medication. At the follow-up visits, a constant (10 mm Hg for systolic blood pressure and 5 mm Hg for diastolic blood pressure) was added to the blood pressure of participants using antihypertensive medication to correct for the effect of treatment on blood pressure levels. This is an established method to adjust for medication use that has less bias and greater power than other methods (27).

### Other variables

Participant sociodemographic and covariate information (age, sex, education, study center, BMI, smoking status, drinking status, prediabetes status, and eGFR) was obtained from SHFS baseline questionnaires that included standardized interviews, medication reviews, and physical examinations as detailed previously (28,29). Prediabetes was defined according to the 1997 American Diabetes Association criteria for impaired fasting glucose tolerance (blood glucose level 110–125 mg/dL) (30). eGFR was calculated by using the Chronic Kidney Disease Epidemiology Collaboration equation (31).

### Statistical analysis

We compared participant baseline characteristics of those with and without incident hypertension and across quartiles of urinary uranium concentrations. We described baseline characteristics, including age (years; continuous), sex (male, female), study center (Arizona, Oklahoma, North Dakota, and South Dakota), education (<12, 12, >12 y), smoking status (never, ever, current), alcohol status (never, ever, current), BMI (continuous), prediabetes status (normal fasting glucose, impaired fasting glucose), systolic blood pressure (mm Hg; continuous), diastolic blood pressure (mm Hg; continuous), eGFR (mL/min; continuous) between those with and without incident hypertension and across quartiles of urinary uranium concentrations. To test group differences, we used Kruskal–Wallis tests for continuous values and χ^2^ tests for categorical variables.

We jointly assessed the prospective association of baseline urinary uranium concentrations with incident hypertension by using a modified Poisson regression with robust variance and the prospective association between baseline urinary uranium concentrations and changes in blood pressure levels measured at follow-up versus baseline by using linear regression (32). To address the lack of independence among family members in the SHFS, we used generalized estimating equations (GEEs). In the main analysis, we estimated the risk ratio (RR) and 95% CIs for incident hypertension. As measured in urine, we calculated the association of uranium exposure with incident hypertension per interquartile range (IQR) increase, quartiles, and with restricted cubic splines. We determined the mean difference (95% CI) for the change in blood pressure levels between baseline and follow-up by baseline urinary uranium levels.

Urinary uranium was right-skewed and log-transformed for analysis. To assess normality assumptions, we used Q–Q plots and kernel density plots. We modeled urinary uranium concentrations as quartiles, continuous log-transformed (and reported per IQR), and restricted cubic splines (knots at 10th, 50th, and 90th percentiles) to allow for flexibility in the dose–response. We used a priori knowledge to make progressive adjustments for available variables associated with hypertension, blood pressure, and uranium. Model 1 was adjusted for age, sex, study center, and smoking status. Model 2 was further adjusted for eGFR, prediabetes status, and BMI. Model 3 was further adjusted for log-transformed urinary arsenic (μg/g creatinine) and cadmium (μg/g creatinine). We analyzed possible effect modification by study center by stratifying by study center. Uranium exposure varied by study center, so we assessed possible effect modification for all main analyses, with adjustment for confounders included up to Model 3 (except study center). We obtained *P* values for interactions by using Wald tests for multiple coefficients. As a sensitivity analysis, we repeated our main models, adjusting for specific gravity instead of standardizing urinary uranium by urinary creatinine because creatinine is affected by kidney function and uranium is nephrotoxic.

## Results

The median age of study participants was 34.1 years; 38.2% of participants were male (Table 1). Median (IQR) urinary uranium concentration was 0.029 µg/g (0.014–0.059). Of the 1,453 participants without hypertension at baseline, 253 (17.4%) developed hypertension during follow-up (mean age, 41.5 y). Compared with participants who did not develop hypertension during follow-up, those who developed hypertension were significantly more likely to be older, be male, self-report as an ever alcohol user, have a higher BMI, have impaired fasting glucose, and have lower eGFR. Median (IQR) levels of urinary uranium were higher among participants from Arizona (0.04 [0.02–0.07] µg/g), and North Dakota and South Dakota (0.04 [0.02–0.08] µg/g) than among participants from Oklahoma (0.02 [0.01–0.03] µg/g). Participants with lower education levels and those with higher eGFR levels were more likely to have higher urinary uranium levels (Table 2).

**Table 1:** Characteristics of Participants at Baseline (Visit 3 Pilot and Visit 4 Combined, 1998–2003), by Hypertension Status at Follow-Up (Visit 5, 2006–2009), Strong Heart Family Study

Characteristic	Overall at baseline^a^	Hypertension status at follow-up		
		No hypertension	Hypertension^b^	*P* value^c^
**No. (%)**	1,453 (100.0)	1,200 (82.6)	253 (17.4)	—
**Age, mean (SD), y**	34.1 (14.0)	32.6 (13.3)	41.5 (14.8)	<.001
**Sex, no. (%)**				
Female	898 (61.8)	771 (64.2)	127 (50.2)	<.001
Male	555 (38.2)	429 (35.8)	126 (49.8)	
**Study center, no. (%)**				
Arizona	162 (11.1)	135 (11.2)	27 (10.7)	.94
Oklahoma	557 (38.3)	461 (38.4)	96 (37.9)	
North Dakota and South Dakota	734 (50.5)	604 (50.3)	130 (51.4)	
**Years of education, no. (%)**				
<12	465 (32.0)	390 (32.5)	75 (29.6)	.66
12	519 (35.7)	424 (35.3)	95 (37.5)	
>12	469 (32.3)	386 (32.2)	83 (32.8)	
**Smoking status, no. (%)**				
Never smoker	589 (40.5)	499 (41.6)	90 (35.6)	.21
Ever smoker	275 (18.9)	223 (18.6)	52 (20.6)	
Current smoker	589 (40.5)	478 (39.8)	111 (43.9)	
**Alcohol status, no. (%)^d^**				
Never drinker	161 (11.1)	140 (11.7)	21 (8.3)	.006
Ever drinker	352 (24.3)	272 (22.7)	80 (31.7)	
Current drinker	938 (64.6)	787 (65.6)	151 (59.9)	
**BMI^e^**	30.1 (7.4)	29.6 (7.1)	32.3 (8.3)	<.001
**Prediabetes status, no. (%)^f^**				
Normal fasting glucose	1,130 (77.8)	961 (80.1)	169 (66.8)	<.001
Impaired fasting glucose	323 (22.2)	239 (19.9)	84 (33.2)	
**Blood pressure, mean (SD), mm Hg **				
Systolic^g^	116.0 (10.7)	114.4 (10.2)	123.5 (9.3)	<.001
Diastolic^h^	73.5 (8.9)	72.5 (8.8)	78.1 (7.9)	<.001
**Urinary uranium,** **µg/g creatinine, median (IQR)**	0.029 (0.014–0.059)	0.029 (0.014–0.058)	0.030 (0.016 vs. 0.066)	.17
**eGFR**	122.0 (16.9)	123.4 (16.5)	115.1 (17.3)	<.001

Abbreviations: BMI, body mass index; eGFR, estimated glomerular filtration rate.

a People with prevalent hypertension at baseline were excluded from analysis.

b Meets criteria for hypertension: having systolic blood pressure 140 mm Hg OR diastolic blood pressure 90 mm Hg OR taking hypertension medication.

c *P* values were determined by Kruskal–Wallis test for continuous variables (age, BMI, blood pressure, urinary uranium, and eGFR) and χ^2^ test for categorical variables (sex, study center, years of education, smoking status, alcohol status, and prediabetes status).

d Two participants did not answer the survey question.

e Calculated as weight in kilograms divided by height in meters squared.

f Normal fasting glucose defined as having fasting blood glucose <110 mg/dL AND no diabetes treatment; impaired fasting glucose defined as having blood glucose level 110–125 mg/dL (30).

g Calculated as sum of first and second measured systolic blood pressure divided by 2.

h Calculated as sum of first and second measured diastolic blood pressure divided by 2.

**Table 2:** Characteristics of Participants Without Hypertension at Baseline (N = 1,453), by Quartile of Baseline Urinary Uranium Level, Strong Heart Family Study, 1998–2009

Characteristic	Overall: 0–6.2 μg/g	Quartile of urinary uranium, μg/g of creatinine				
		Quartile 1: <0.01	Quartile 2: 0.01–0.03	Quartile 3: 0.03–0.06	Quartile 4: >0.06	*P* value^a^
**No. (%)**	1,453 (100.0)	345 (23.7)	372 (25.6)	364 (25.0)	372 (25.6)	—
**Age, mean (SD), y**	34.1 (14.0)	34.5 (14.2)	33.9 (14.0)	33.9 (13.6)	34.2 (14.3)	.80
**Sex, no. (%)**						
Female	898 (61.8)	223 (64.6)	225 (60.5)	215 (59.1)	235 (63.2)	.41
Male	555 (38.2)	122 (35.4)	147 (39.5)	149 (40.9)	137 (36.8)	
**Study center, no. (%)**						
Arizona	162 (11.1)	19 (5.5)	35 (9.4)	56 (15.4)	52 (14.0)	<.001
Oklahoma	557 (38.3)	199 (57.7)	177 (47.6)	118 (32.4)	63 (16.9)	
North Dakota and South Dakota	734 (50.5)	127 (36.8)	160 (43.0)	190 (52.2)	257 (69.1)	
**Years of education, no. (%)**						
<12	465 (32.0)	91 (26.4)	109 (29.3)	129 (35.4)	136 (36.6)	.04
12	519 (35.7)	130 (37.7)	134 (36.0)	124 (34.1)	131 (35.2)	
>12	469 (32.3)	124 (35.9)	129 (34.7)	111 (30.5)	105 (28.2)	
**Smoking status, no. (%)**						
Never smoker	589 (40.5)	160 (46.4)	155 (41.7)	140 (38.5)	134 (36.0)	.09
Ever smoker	275 (18.9)	62 (18.0)	72 (19.4)	74 (20.3)	67 (18.0)	
Current smoker	589 (40.5)	123 (35.7)	145 (39.0)	150 (41.2)	171 (46.0)	
**Alcohol status, no. (%)^b^**						
Never drinker	161 (11.1)	34 (9.9)	40 (10.8)	48 (13.2)	39 (10.5)	.54
Ever drinker	352 (24.3)	94 (27.2)	92 (24.9)	84 (23.1)	82 (22.0)	
Current drinker	938 (64.6)	217 (62.9)	238 (64.3)	232 (63.7)	251 (67.5)	
**BMI, mean (SD)^c^**	30.1 (7.4)	30.1 (7.0)	30.1 (7.0)	30.3 (8.0)	29.9 (7.6)	.83
**Prediabetes status^d^**						
Normal fasting glucose	1,130 (77.8)	267 (77.4)	301 (80.9)	277 (76.1)	285 (76.6)	.37
Impaired fasting glucose	323 (22.2)	78 (22.6)	71 (19.1)	87 (23.9)	87 (23.4)	
**eGFR, mean (SD)**	122.0 (16.9)	121.1 (17.5)	120.5 (16.7)	123.3 (16.4)	123.0 (16.9)	.04
**Blood pressure, mean (SD), mm Hg**						
Systolic^e^	116.0 (10.7)	115.5 (10.6)	116.8 (10.8)	115.9 (10.7)	115.7 (10.5)	.94
Diastolic^f^	73.5 (8.9)	73.2 (9.3)	73.5 (9.0)	73.8 (9.0)	73.4 (8.3)	.68

Abbreviations: BMI, body mass index; eGFR, estimated glomerular filtration rate.

a *P* values were determined by Kruskal–Wallis test for continuous variables (age, BMI, blood pressure, urinary uranium, and eGFR) and χ^2^ test for categorical variables (sex, study center, years of education, smoking status, alcohol status, and prediabetes status).

b Two participants did not answer the survey question.

c Calculated as weight in kilograms divided by height in meters squared.

d Normal fasting glucose defined as having fasting blood glucose <110 mg/dL AND no diabetes treatment; impaired fasting glucose defined as having blood glucose level 110–125 mg/dL (30).

e Calculated as sum of first and second measured systolic blood pressure divided by 2.

f Calculated as sum of first and second measured diastolic blood pressure divided by 2.

### Incident hypertension

In the fully adjusted models, the RRs (95% CI) for incident hypertension for the second, third, and fourth quartiles of urinary uranium compared with the first quartile were 1.31 (0.96–1.78), 1.32 (0.95–1.83), and 1.44 (1.04–1.99) in the fully adjusted model (Table 3, Model 3), including adjustment for arsenic and cadmium. The RR (95% CI) for incident hypertension comparing the 25th and 75th percentiles was 1.15 (0.99–1.33) (Table 3). Uranium remained associated with incident hypertension in flexible dose–response models (Figure). In stratified models by study center, the RRs (95% CI) per IQR of urinary uranium were 1.01 (0.64–1.59) for Arizona, 1.25 (0.96–1.61) for Oklahoma, and 1.06 (0.88– 1.28) for North Dakota and South Dakota (*P* value for interaction = .55).

**Table 3:** Risk Ratios (RRs) for Incident Hypertension, by Quartile Increase in Urinary Uranium Among Participants (N = 1,453), Strong Heart Family Study, 1998–2009^a^

Model	Comparison of 25th and 75th percentiles (0.01 vs 0.06 μg/g)	Quartile increase in urinary uranium, μg/g of creatinine			
		Quartile 1: <0.01	Quartile 2: 0.01–0.03	Quartile 3: 0.03–0.06	Quartile 4: >0.06
No. of cases^b^/no. of noncases	253/1,200	52/312	66/297	66/297	69/294
Model 1, RR (95% CI)^c^	1.11 (0.96–1.28)	1 [Reference]	1.27 (0.94–1.72)	1.26 (0.92–1.74)	1.34 (0.96–1.86)
Model 2, RR (95% CI)^d^	1.13 (0.97–1.31)	1 [Reference]	1.28 (0.95–1.74)	1.29 (0.93–1.77)	1.38 (1.00–1.91)
Model 3, RR (95% CI)^e^	1.15 (0.99–1.33)	1 [Reference]	1.31 (0.96–1.78)	1.32 (0.95–1.83)	1.44 (1.04–1.99)

a Models were estimated by Poisson regression with robust error variance using generalized estimating equations with an independent covariance accounting for family clustering.

b Cases of hypertension were defined as systolic blood pressure ≥140 mm Hg OR diastolic blood pressure ≥90 mm Hg OR taking hypertension medication.

c Model 1 adjusted for age (continuous), sex (male/female), study center (Arizona/Oklahoma/South Dakota and North Dakota), and smoking status (never/former/current).

d Model 2 further adjusted for estimated glomerular filtration rate (continuous), prediabetes status (normal fasting glucose/impaired fasting glucose), and body mass index (continuous).

e Model 3 further adjusted for log-transformed arsenic (continuous) and log-transformed cadmium (continuous).

**Figure:**
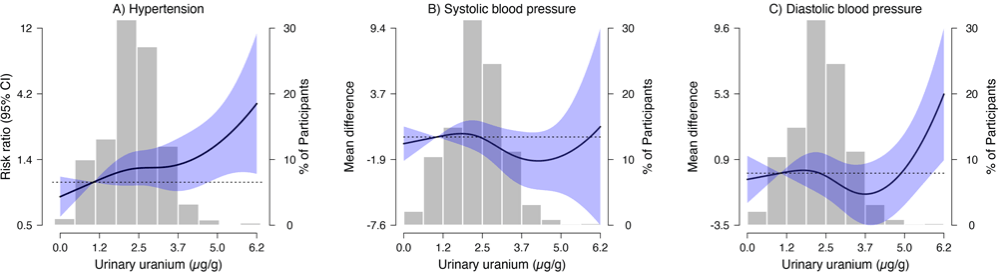
Risk ratio (RR) of hypertension (A) and mean difference (95% CI) for the change in systolic and diastolic blood pressure (mm Hg) levels at follow-up versus baseline (B, C) per log urinary uranium (μg/g creatinine) (N = 1,453), Strong Heart Family Study 1998–2009. The solid black line indicates adjusted effect estimate; shading indicates 95% CIs. Effect estimates were calculated by using restricted cubic splines for uranium with knots at the 10th (referent), 50th, and 90th percentiles of the urinary uranium (μg/g creatinine) distribution, and adjusted for sex, age, smoking status, study center, eGFR, prediabetes status, log urinary arsenic, and log urinary cadmium. Models include generalized estimating equations (GEEs) to account for the clustering of participants within families. Histograms indicate the distribution of log-transformed urinary uranium levels. Incident hypertension was defined as having a systolic blood pressure ≥140 mm Hg OR diastolic blood pressure ≥90 mm Hg OR taking hypertension medication. The horizontal dashed line indicates no association between urinary uranium and the outcomes.

### Blood pressure

In the fully adjusted models, the mean difference (95% CI) for the change in systolic blood pressure at follow-up versus baseline per IQR of urinary uranium was −0.02 (−0.04 to 0.01) mm Hg, with evidence of nonlinearity for quartiles 2, 3 and 4 compared with the lowest quartile (Table 4, Model 3). We found no significant association between urinary uranium and diastolic blood pressure, and results followed a similar direction as for systolic blood pressure (Table 4). In models with flexible splines, the associations of urinary uranium with systolic blood pressure and diastolic blood pressure were nonlinear, with a potential increase in systolic blood pressure and particularly diastolic blood pressure at follow-up versus baseline at higher baseline urinary uranium levels (Figure).

**Table 4:** Mean Differences in Change in Systolic and Diastolic Blood Pressure Levels at Follow-Up vs Baseline, by Quartile of Baseline Urinary Uranium Level Among Participants (N = 1,453), Strong Heart Family Study, 1998–2009^a^

Model	IQR (0.01 vs 0.06 μg/g)	Quartile of urinary uranium, μg/g of creatinine			
		Quartile 1: <0.01	Quartile 2: 0.01–0.03	Quartile 3: 0.03–0.06	Quartile 4: >0.06
**No. (%)**	1,453 (100.0)	364 (25.0)	363 (24.9)	363 (24.9)	363 (24.9)
**Systolic blood pressure, mm Hg**					
Model 1, β (95% CI)^b^	−0.02 (−0.05 to 0.00)	1 [Reference]	0.08 (−1.67 to 1.84)	−0.30 (−2.04 to 1.44)	−1.94 (−3.78 to −0.10)
Model 2, β (95% CI)^c^	−0.02 (−0.05 to 0.00)	1 [Reference]	0.08 (−1.70 to 1.85)	−0.35 (−2.08 to 1.38)	−2.00 (−3.83 to −0.16)
Model 3, β (95% CI)^d^	−0.02 (−0.04 to 0.01)	1 [Reference]	0.24 (−1.55 to 2.02)	−0.04 (−1.84 to 1.75)	−1.48 (−3.32 to 0.37)
**Diastolic blood pressure, mm Hg**					
Model 1, β (95% CI)^b^	−0.01 (−0.04 to 0.01)	1 [Reference]	0.50 (−0.99 to 1.99)	−0.66 (−2.39 to 1.07)	−1.49 (−3.33 to 0.35)
Model 2, β (95% CI)^c^	−0.02 (−0.04 to 0.01)	1 [Reference]	0.43 (−1.04 to 1.90)	−0.68 (−2.40 to 1.03)	−1.53 (−3.34 to 0.28)
Model 3, β (95% CI)^d^	−0.01 (−0.03 to 0.01)	1 [Reference]	0.39 (−1.02 to 1.80)	−0.60 (−2.25 to 1.06)	−1.25 (−2.95 to 0.44)

a Mean differences in blood pressure level were estimated by generalized estimating equations with an independent covariance accounting for family clustering.

b Model 1 adjusted for age (continuous), sex (male/female), study center (Arizona/Oklahoma/South Dakota and North Dakota), and smoking status (never/former/current).

c Model 2 further adjusted for estimated glomerular filtration rate (continuous), prediabetes status (normal fasting glucose/impaired fasting glucose), and body mass index (continuous).

d Model 3 further adjusted for log-transformed arsenic (continuous) and log-transformed cadmium (continuous).

We observed differences in the association between urinary uranium and blood pressure by study center. In stratified models by study center, the change in systolic blood pressure at follow-up versus baseline per IQR of urinary uranium was inverse in Arizona and Oklahoma and positive in North Dakota and South Dakota at higher ends of the uranium exposure distribution. The change in diastolic blood pressure at follow-up versus baseline per IQR of urinary uranium was inverse in Arizona and positive in Oklahoma and North Dakota and South Dakota at higher ends of the uranium exposure distribution. Corresponding fully adjusted mean differences (95% CI) for the change in systolic blood pressure at follow-up versus baseline per IQR of urinary uranium were −0.04 (−0.14 to 0.05) mm Hg for Arizona, 0.01 (−0.01 to 0.03) mm Hg for Oklahoma, and −0.04 (−0.07 to 0) mm Hg for North Dakota and South Dakota *(P* value for interaction = .03).

### Sensitivity analyses

We observed similar and stronger findings when adjusting for specific gravity instead of standardizing urinary uranium for urinary creatinine. In fully adjusted models, the RR (95% CI) of hypertension comparing the 75th and 25th percentiles was 1.23 (1.05–1.44). The mean difference (95% CI) for the change in systolic blood pressure at follow-up versus baseline per IQR of urinary uranium was −0.01 (−0.05 to 0.02) mm Hg. Results were similar for urinary uranium and diastolic blood pressure compared with urinary creatinine standardization. In flexible spline dose–response plots, the association of urinary uranium with systolic blood pressure was linear and inverse at higher baseline urinary uranium levels.

## Discussion

In the SHFS, conducted with American Indian communities in the Southwest and the Great Plains, urinary uranium was associated with a moderately increased risk for hypertension. The dose–response was linear for hypertension, null for the change in systolic blood pressure, and nonlinear for the change in diastolic blood pressure, which showed a positive association only beyond 5 µg U/g creatinine. We observed effect measure modification by study center. The association of urinary uranium with incident hypertension was stronger in Oklahoma than in Arizona or North Dakota and South Dakota. The change in systolic blood pressure levels at follow-up versus baseline per IQR of urinary uranium was inverse in Arizona and Oklahoma, and positive in North Dakota and South Dakota at the higher ends of the urinary uranium distribution.

Few studies have evaluated the relationship between uranium and hypertension or blood pressure. Prior literature evaluated uranium as a component of metal mixtures and suggested that uranium acts additively with lead and may affect waist circumference (33). Urinary uranium (> 0.028 µg/L) has also been associated with 30% higher odds of prevalent type 2 diabetes (34). Our results were consistent with a study that found a positive association between uranium and hypertension across 3 NHANES survey cycles (2012–2016) (17). Conversely, urinary uranium was not associated with hypertension in a larger study that used 9 survey cycles (1999–2016), although that study dichotomized urinary uranium into low and high categories without leveraging the full distribution, and models were unadjusted (34). Another study, which examined the health of residents living near an old uranium mine, found no association between uranium exposure, assessed via residential history, and hypertension (35). In a cohort of pregnant study participants in California, uranium in drinking water was inversely associated with hypertensive disorders in pregnancy (36). The current understanding of the mechanism by which uranium exerts its chemical toxicologic effects is limited (37). In a small study (N = 193 participants) in a community chronically exposed to low-to-moderate uranium levels in drinking water (median [IQR], 25 [5–148] µg/L) in Finland, higher urinary uranium levels were associated with higher systolic and diastolic blood pressure levels (38). To our knowledge, our study is novel in its prospective associations between urinary uranium with both hypertension and blood pressure and supports that uranium exposure is associated with a higher risk of hypertension and higher blood pressure levels.

The overall association between baseline urinary uranium and the change in blood pressure from baseline to follow-up differed across study centers. In stratified analyses, the association of continuous log-transformed urinary uranium with the change in systolic blood pressure was inverse in all 3 study centers at levels below 4.5 µg U/g creatinine. In North Dakota and South Dakota, the only study center where urinary uranium exceeded 4.5 µg U/g creatinine, associations were positive above 4.5 µg U/g creatinine. For the association of urinary uranium with the change in diastolic blood pressure, associations were positive in Oklahoma and in North Dakota and South Dakota at higher levels of the urinary uranium distribution. The positive association of urinary uranium with both hypertension and the change over time in diastolic blood pressure in the 3 SHS centers was strongest in Oklahoma, the center with overall lower levels of urinary uranium (39). A possible explanation could be related to regional differences in other environmental exposures that may either overwhelm or modify the association between uranium and hypertension that were not captured in our dataset. For example, arsenic and uranium frequently co-occur in both drinking water sources and urine in SHFS communities (1,7,40). Hence, our effect estimates for uranium with the change in blood pressure levels were attenuated with further adjustment for arsenic and cadmium, which are established risk factors for CVD (41). While self-reported dietary patterns and the association between food groups and urinary uranium differ across SHFS study sites, prior work indicates that diet explains relatively little variability in urinary uranium concentrations (dust exposure could also be relevant for some SHFS communities) (39). More research is needed to disentangle these inconsistencies between the association with blood pressure overall and by study center.

While this analysis provides useful insights into the associations of chronic uranium exposure with blood pressure and hypertension, there are several limitations. First, because most uranium is quickly excreted from the body, the measurement may not reflect the actual chronic exposure of participants to uranium (42). However, uranium levels in drinking water tend to be stable, so we expect that urinary uranium levels are likely to reflect chronic exposures, if there is no change in the source of drinking water. We were unable to evaluate if drinking water source changed over time because drinking water source was not collected from SHFS participants. Furthermore, it is possible that those with the highest levels of uranium exposure may have already developed hypertension before baseline, and thus were excluded from analyses, resulting in selection bias. We were unable to adjust for lead exposure in this analysis because blood lead was not measured from samples collected at the baseline SHFS visit. Metal exposures are correlated in the SHFS, so future studies should explore complex metal mixtures to identify the effects of the most toxic metal components on blood pressure, hypertension, and other critical CVD risk factors, which was beyond the scope of this analysis. Additionally, there remains a critical need to evaluate uranium exposure with kidney disease events, subclinical measures, and risk factors, as these analyses were beyond the scope of this study.

We observed consistent, although stronger, effect estimates when adjusting for specific gravity compared with when standardizing urinary uranium for urinary creatinine. Uranium is nephrotoxic, as demonstrated in animal studies, and may influence the excretion of metals (including U) in urine (43). While we adjusted for eGFR to account for kidney function, it is unknown if models adjusting for specific gravity are more appropriate for studies of urinary uranium and cardiometabolic outcomes. Future studies can use environmental monitoring to avoid reverse causality concerns.

Prior SHS and SHFS work found arsenic, cadmium, and lead as risk factors for CVD, consistent with established evidence (41). For uranium, however, not enough research exists to make a comprehensive determination. Our findings, in one of the first prospective studies available, support an association consistent with previous cross-sectional studies, but more work is needed. Our research is especially relevant for American Indian and Alaska Native communities, in which disparities in uranium exposure, hypertension, and CVD are well-established. Future studies should evaluate the role of low-dose chronic uranium exposure on hypertension, elevated blood pressure levels, and other CVD risk factors, as well as clinical CVD in larger nationwide cohorts to better understand the relationship over time, including potential nonlinear patterns. Mechanistic and experimental research and understanding the processes by which uranium negatively affects biologic processes will also lend insight into how to prevent and treat diseases associated with uranium exposure. Furthermore, additional evidence could have implications for primary and secondary interventions. For example, community interventions and federal regulations (eg, the Final Arsenic Rule by the US Environmental Protection Agency in 2001 [44] reduced the maximum contaminant level from 50 µg/L to 10 µg/L) have been successful in reducing water arsenic exposure (45). Uranium exposure through drinking water, soil, and food is widespread in the US, particularly in western states. Recent evidence suggests drinking water is a substantial source of uranium exposure in SHS communities (5). Similar strategies as those developed for arsenic might be necessary to reduce uranium in drinking water. Clinical care settings could be used as an additional screening tool to identify patients who obtain drinking water from wells known to have high levels of uranium. Reducing uranium in drinking water can reduce disparities in exposure and related health outcomes.
